# Protein kinase R-like ER kinase (PERK) is involved in the endoplasmic reticulum stress response of its psyllid vector to *Candidatus Liberibacter solanacearum* infection

**DOI:** 10.1128/spectrum.03025-25

**Published:** 2025-12-26

**Authors:** Ola Jassar, Murad Ghanim

**Affiliations:** 1 Department of Entomology, Volcani Institute 530612 , Rishon LeZion, Israel; 2 The Robert H. Smith Faculty of Agriculture, Food & Environment, the Hebrew University of Jerusalem 108407 , Rehovot, Israel; Connecticut Agricultural Experiment Station, New Haven, Connecticut, USA

**Keywords:** Liberibacter, Psyllid, ER stress, UPR, PERK, apoptosis

## Abstract

Vector-borne transmission of plant and animal pathogens requires active engagement of the vector’s immune system, as pathogens must overcome barriers and exploit host cellular mechanisms. *Candidatus Liberibacter solanacearum* (CLso) and *C. L. asiaticus* (CLas) are gram-negative, phloem-limited bacteria transmitted by the psyllids *Bactericera trigonica* and *Diaphorina citri*, respectively. Both pathogens induce endoplasmic reticulum (ER) stress and apoptosis in the midguts of their respective vectors, promoting their transmission. The protein kinase R-like endoplasmic reticulum kinase (PERK), a key regulator of ER stress and the unfolded protein response (UPR), plays a crucial role in modulating immunity. In this study, we investigated the role of PERK in CLso-induced apoptosis. PERK expression was downregulated in CLso-infected adult psyllids and midguts, yet immunostaining revealed a significant increase in phosphorylated (active) PERK in infected midguts, despite reduced gene expression. Additionally, ER stress-inducing treatments in both infected and uninfected psyllids demonstrated that CLso infection differentially regulates PERK expression. Interestingly, similar treatments in CLas-infected and uninfected *D. citri* yielded different results, suggesting species-specific immune responses. Furthermore, inhibition of PERK altered the expression of apoptosis-related genes in CLso-infected psyllids, indicating its involvement in CLso-induced apoptosis. Our findings suggest that PERK and UPR play a role in initiating immune responses at the ER interface in response to CLso, ultimately contributing to apoptosis, which may aid pathogen transmission. While the precise mechanisms by which CLso controls these processes remain unclear, this study provides new insights into how vector-borne plant and potentially animal pathogens manipulate host cellular pathways to enhance their spread.

**IMPORTANCE:**

This study provides valuable insights into how vector-borne pathogens manipulate host cellular pathways to promote their survival and transmission. *Candidatus Liberibacter solanacearum* (CLso) and *C. L. asiaticus* (CLas) cause severe plant diseases, such as zebra chip in potatoes, carrot yellows, and huanglongbing (citrus greening), posing significant threats to global agriculture. By revealing the role of PERK and the unfolded protein response (UPR) in CLso-induced apoptosis, our findings contribute to the growing understanding of insect immunity and pathogen-host interactions. Understanding how CLso influences ER stress and immune signaling in its psyllid vector could lead to innovative strategies to disrupt pathogen persistence and transmission, ultimately supporting disease management efforts.

## INTRODUCTION

Vector-mediated persistent transmission of plant and animal pathogens by insects involves complex interactions between the pathogen and the insect proteins (1, 2). It further depends on the ability of the pathogen to overcome the immunity of the insect and breach cellular membranes for transmission (3–5). Although such interactions are important and these pathogens pose a continuous threat to human health, agricultural products, and more, little is known about the molecular mechanisms by which vector-borne pathogens overcome vector immunity. The innate immune responses in insect vectors play a critical role in the dynamics of pathogen infection in their hosts and in pathogen movement between cells and tissues during the transmission process (6, 7), during which pathogens can exploit the host cell machinery and avoid or escape the host immune defenses for efficient transmission (8). The main barrier for pathogen transmission by insect vectors during their persistence and circulation is the midgut. However, the molecular mechanisms that specifically underlay the pathogen presence in the midgut and the gut cellular responses are not well studied.

**Fig 1:**
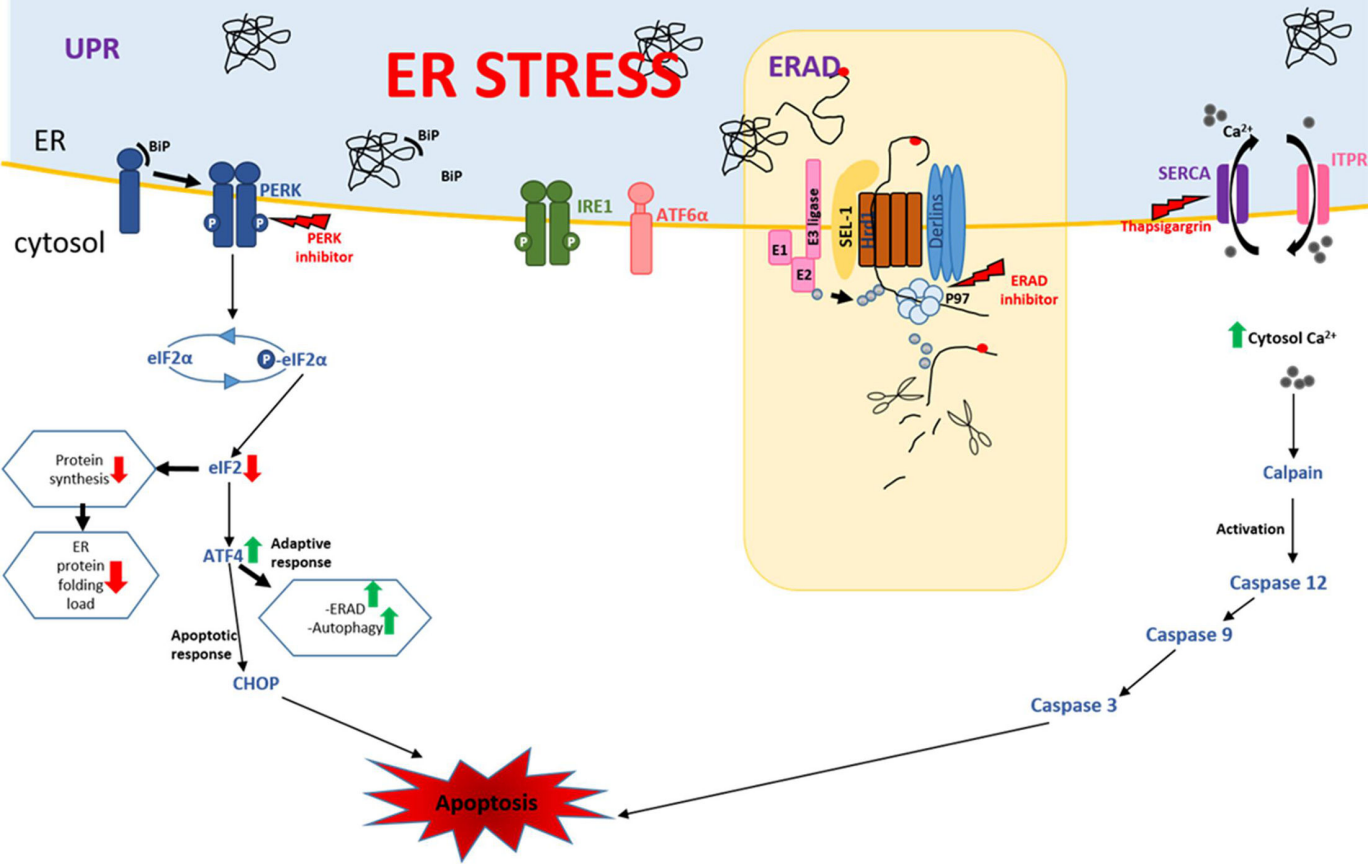
ER stress-induced pathways. The induced UPR pathway, orchestrated by the sensors PERK, ATF6, and IRE1, can lead to an adaptive response or apoptosis. PERK is maintained in its inactive state through its association with BiP. An increase in unfolded proteins in the ER lumen causes the release of BiP from the PERK stress-sensing domain, resulting in PERK activation via auto-phosphorylation and oligomerization. Activated PERK phosphorylates eIF2α in the cytoplasm, resulting in reduced activity of eIF2. ATF4 is increased under conditions of limiting eIF2, reducing global protein synthesis as a mechanism to manage the load of unfolded proteins in the ER. ERAD pathway targets unfolded proteins inside the ER, dislocates them to the cytosol for degradation. Calcium influx (SERCA) pumps and efflux (ITPR) pumps maintain the calcium homeostasis in the ER as well as in the cytosol. Depletion of calcium in the ER induces ER stress, and its accumulation in the cytosol can lead to apoptosis by a cascade of caspases.

The endoplasmic reticulum (ER) is an intracellular organelle that regulates various metabolic activities, such as metabolism of carbohydrates, lipid biogenesis and calcium homeostasis, protein synthesis, and post-translational modification of many secretory and membrane proteins (9, 10). The ER further serves as a sub-cellular compartment involved in the proper folding of proteins after synthesis and their maturation (11). Disrupting ER functions and imbalance of ER homeostasis results in the accumulation of unfolded or misfolded proteins, which causes ER stress and triggers the unfolded protein response (UPR), a coordinated process aimed at restoring protein homeostasis in the ER (12–14). Sensor proteins of the UPR respond to the imbalance between unfolded proteins and binding immunoglobulin protein (BiP) chaperones in the ER lumen. BiP acts as a regulator of UPR by binding to the sensor proteins to keep them inactive under normal conditions. Upon ER stress, BiP dissociates from these sensors, allowing their activation (15–17). UPR response is initiated by three signaling proteins: IRE1α (inositol-requiring protein-1α), PERK (protein kinase RNA [PKR]-like ER kinase), and ATF6 (activating transcription factor 6). These sensors are activated by the accumulation of unfolded proteins in the ER lumen, and each activates a transducer: ATF4 via PERK, cleaved ATF6 by ATF6, and spliced XBP1 by IRE1 (13, 18) (Fig. 1). The combined effects of the activation of these sensors include an upregulation of genes encoding proteins involved in the secretory pathway, which aims to reduce the ER stress by shuttling unfolded proteins outside the ER lumen for degradation by the ER-associated degradation (ERAD) mechanism (19). UPR increases the ER protein-folding capacity, reduces global protein synthesis, and enhances the ERAD of misfolded proteins (Fig. 1) (20). In the case of a prolonged ER stress or when the adaptive response fails to cope with the stress, UPR signaling may initiate apoptotic (cell death) responses (21–23). ER-induced apoptosis occurs via three primary pathways, including the IRE1/ASK1/JNK pathway, the caspase-12 kinase pathway, and the PERK/ATF4/CHOP pathway (21, 22, 24, 25). PERK typically responds to ER stress through dissociation of BiP from PERK, followed by autophosphorylation, homomultimerization (dimerization), and activation. Active PERK phosphorylates the α-subunit of the translation initiation factor eIF2 (eIF2α) at Ser51, resulting in the attenuation of global translation initiation and reducing the load of newly synthesized proteins, many of which are destined to enter the already stressed ER lumen (12, 20). If the stress in the ER is not alleviated, eIF2α promotes transcription of ATF4, which converges on the promoters of target genes, including C/EBP homologous protein (CHOP) (Fig. 1). PERK/ATF4/CHOP signaling pathway plays a pivotal role in inducing apoptosis (18, 26, 27). Although all three pathways are usually activated by any ER stress event, the timing of their activation can differ (28, 29). IRE1α and ATF6 pathway activities can be attenuated compared to the PERK pathway during the apoptotic phase of prolonged ER stress (29, 30).

ER stress-induced apoptosis can play a crucial role in interactions between vectors and vector-borne bacterial species. Preventing or delaying apoptosis provides a survival advantage because it facilitates bacterial replication inside the host cells (31). *Candidatus Liberibacter solanacearum* (CLso) haplotype D is a gram-negative, phloem-limited bacterium transmitted by the carrot psyllid *Bactericera trigonica* in Israel. Similar to *Candidatus Liberibacter asiaticus* (CLas), the causative agent of citrus greening disease (32), CLso is transmitted by psyllids in a persistent, propagative manner and is associated with serious diseases of solanaceous crops, such as tomato and potato, and apiaceous vegetables, including carrots, celery, fennel, and parsley (33). After acquisition by the psyllid during feeding, CLso moves along the digestive system and colonizes the midgut (34). After replicating in the midgut, CLso crosses into the hemolymph, circulates, and infects other insect tissues, including the salivary glands, from which it is injected into the new plant with salivary secretions (35, 36). Previous studies have shown that CLas and CLso are associated with the ER of their psyllid vector midguts, and it has been confirmed that both induce ER stress in midgut cells (19, 37, 38). Additionally, we have recently shown that CLso induces ERAD in its psyllid midguts (19). This induction is likely to be involved in CLso propagation and transmission. Previous reports have also confirmed that CLas and CLso induce apoptosis in *Liberibacter*-infected midguts (34, 39, 40). In the current study, we further confirmed that upon the induction of apoptosis by CLso in midguts of the carrot psyllid, PERK and its downstream pathways play a major role in the observed induction of apoptosis.

## RESULTS

### CLso induces apoptosis in the midgut of the carrot psyllid

**Fig 2:**
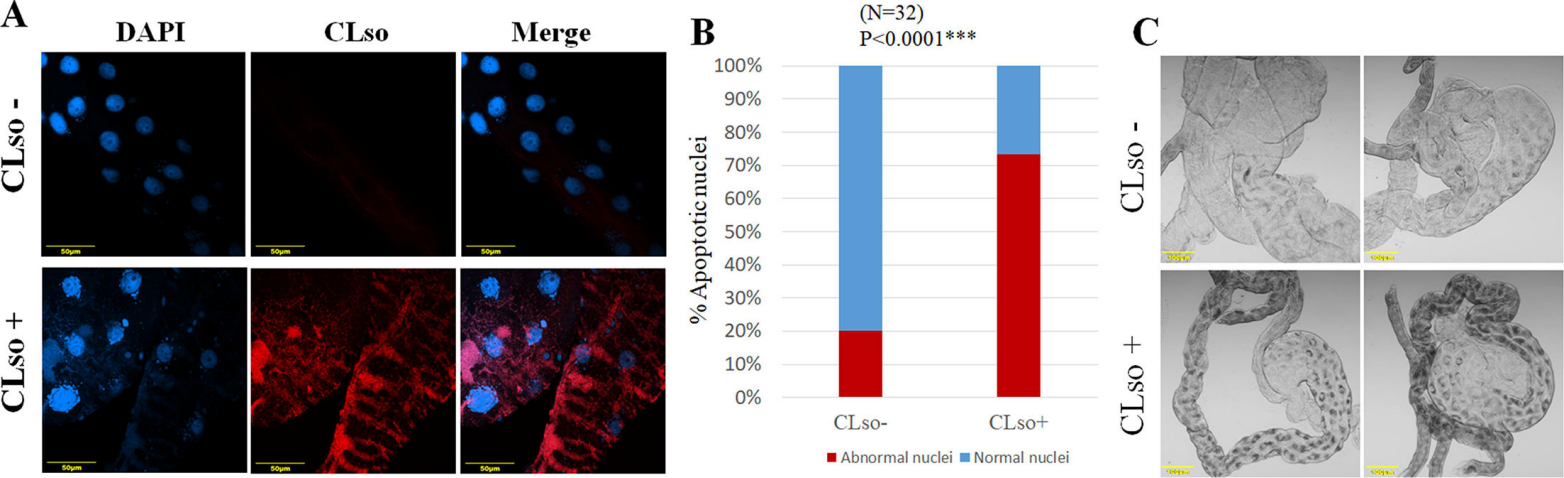
Quantification of normal/abnormal nuclei of midguts dissected from CLso-infected and uninfected psyllids. (**A**) DAPI staining shows irregular nuclei in shape, size, and fragmentation throughout the cytoplasm in CLso-infected midgut. (**B**) Quantification of normal/abnormal nuclei of midguts dissected from CLso-infected and uninfected psyllids. (**C**) Light micrograph of midguts dissected from CLso-infected and uninfected psyllids shows the formation of dark necrotic spots in the infected sample. (***P < 0.0001).

The phenotypic effect of CLso on the psyllid midgut was examined using FISH. Staining the nuclei of midguts of CLso-infected and uninfected psyllids using DAPI showed that the majority of the nuclei in CLso-infected samples are abnormal. They showed shrinkage of the nucleus and loss of their normal elliptical structure, possibly due to irreversible chromatin condensation in adult midguts (Fig. 2A). Additionally, many irregularly shaped and spaced punctate regions of chromatin were observed outside of the nucleus. Bright-field images of psyllid midguts showed that CLso-infected midguts contained black lesions and necrotic spots (Fig. 2C). Other nuclei showed fragmentation of the nucleus with the irregular distribution of chromatin throughout the cell. Nuclei from psyllids reared on healthy plants appeared regularly dispersed in the cells and uniform in shape and size (Fig. 2A). Quantification of this effect showed that 73.3% of the nuclei were abnormal in midguts dissected from CLso-infected adult psyllids, compared to only 20.08% in adult psyllids reared on healthy plants (Fig. 2B). The observed phenotypes, reminiscent of apoptotic responses, were further pursued by investigating the expression of apoptosis-related genes. This was done using whole adults and dissected midguts after CLso acquisition. The results showed that the expression of all inhibitors of apoptosis (IAP) genes was significantly downregulated. However, the apoptosis inducer AIF1 was significantly upregulated only in the midgut samples, but not in whole adults, likely due to the localized effect of CLso in the midgut being diluted when analyzing the entire body (Fig. S1A and B).

### PERK expression is downregulated in CLso-infected psyllids

**Fig 3:**
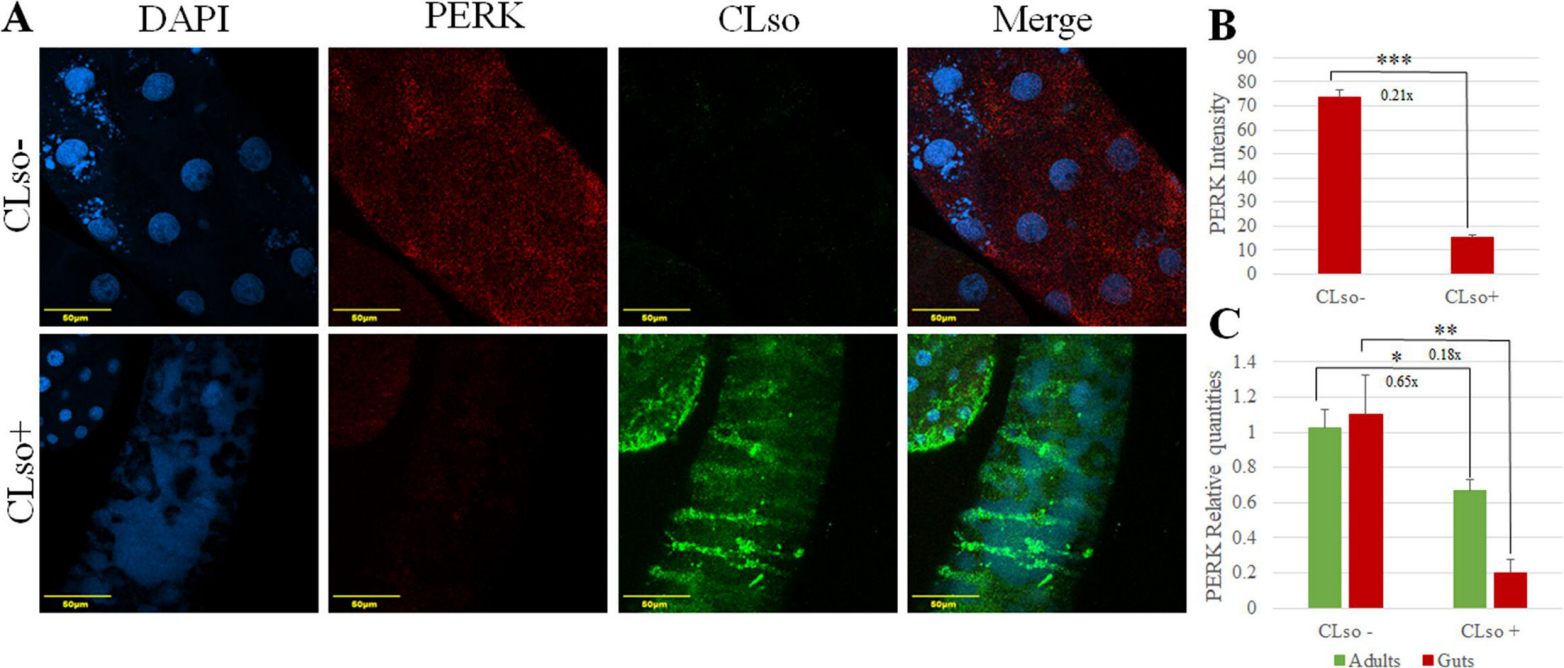
PERK is downregulated in midguts and whole adults of CLso-infected psyllids. (**A**) Co-immunostaining of CLso (green) and PERK (red) in midguts dissected from CLso-infected and uninfected psyllids and counterstained with DAPI (blue), showing that CLso infection resulted in reduced amounts of PERK in the psyllid midgut. (**B**) Significant difference in the intensity of PERK as a result of CLso infection measured using FIJI software. (**C**) Relative quantification of the expression of PERK in midguts and whole adults of CLso-infected and uninfected psyllids. (*P < 0.05, **P < 0.01, ***P < 0.0001).

Among the three main sensors of UPR, sequences of two were identified in the transcriptomic analysis we previously performed on the carrot psyllid (38). The expression of UPR-associated genes was quantified in CLso-infected and uninfected psyllids. The expression of protein kinase R (PKR)-like endoplasmic reticulum kinase (PERK), also known as eukaryotic translation initiation factor 2-alpha kinase 3, was surprisingly downregulated in both CLso-infected psyllids (whole adults) (0.65×, 1.53-fold less) and midguts (0.18×, 5.6-fold less) (Fig. 3C). The relative expressions of the three main genes involved in the PERK pathway in UPR (eIF2α, ATF4, and CHOP) were quantified, and no significant difference was detected (data not shown). To confirm the qRT-PCR results, immunostaining using anti-CLso and anti-PERK antibodies was performed (Fig. 3A). The immunostaining intensity levels were quantified using Fiji software, which further supported the qRT-PCR and immunostaining results, confirming reduced PERK protein levels in CLso-infected midguts (Fig. 3B).

### CLso co-localizes with activated PERK and enhances its expression

**Fig 4:**
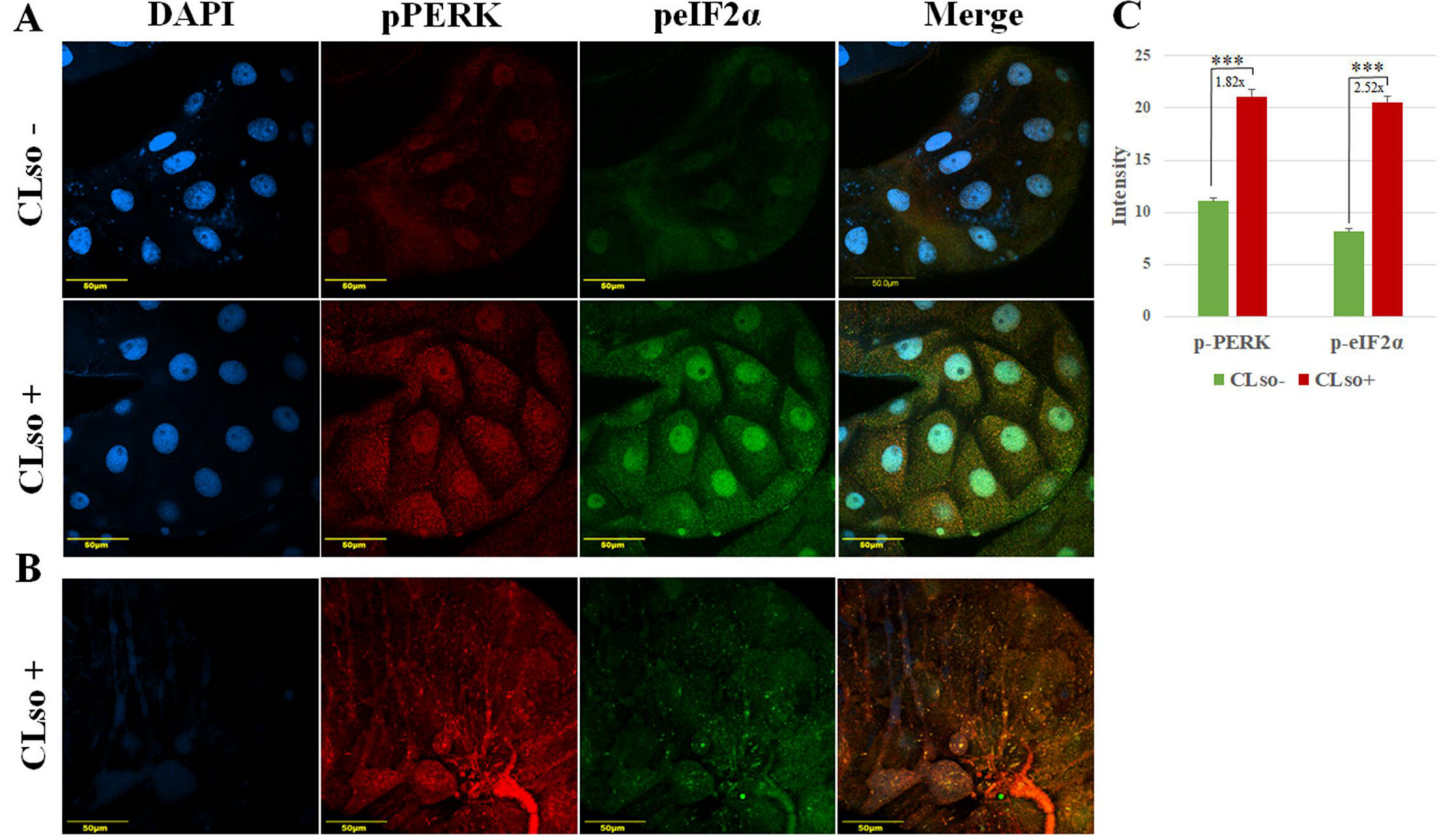
PERK and eIF2α are more active in the midgut cells of CLso-infected psyllids. (**A**) Co-immunostaining of phospho-PERK (red) and phospho-eIF2α (green) in midguts dissected from CLso-infected and uninfected psyllids and counterstained with DAPI (blue). (**B**) Phospho-PERK localizes in stripes pattern at the surface (outer focal plane) in CLso-infected psyllid. (**C**) The average of intensity measurement of phospho-PERK and phospho-eIF2α using FIJI software. (***P < 0.0001).

Following the downregulation of PERK in CLso-infected samples, we examined its expression in the activated form. Immunostaining using specific antibodies for the phosphorylated forms of PERK (phospho-PERK) and eIF2α (phospho-eIF2α) was performed to assess their activity levels in the midguts of CLso-infected and uninfected psyllids. Results showed that both phosphorylated PERK and eIF2α levels were significantly higher in CLso-infected midguts (Fig. 4A). The signal intensities of the two proteins were quantified using FIJI software (Fig. 4C). Figure 4B presents outer focal plane images of a CLso-infected midgut. In this focal plane, phospho-PERK (red) displays a striped pattern resembling the CLso distribution previously shown in Fig. 3A. In contrast, phospho-eIF2α does not follow this pattern.

### ER stress drugs combined with CLso infection differentially influence PERK expression

**Fig 5:**
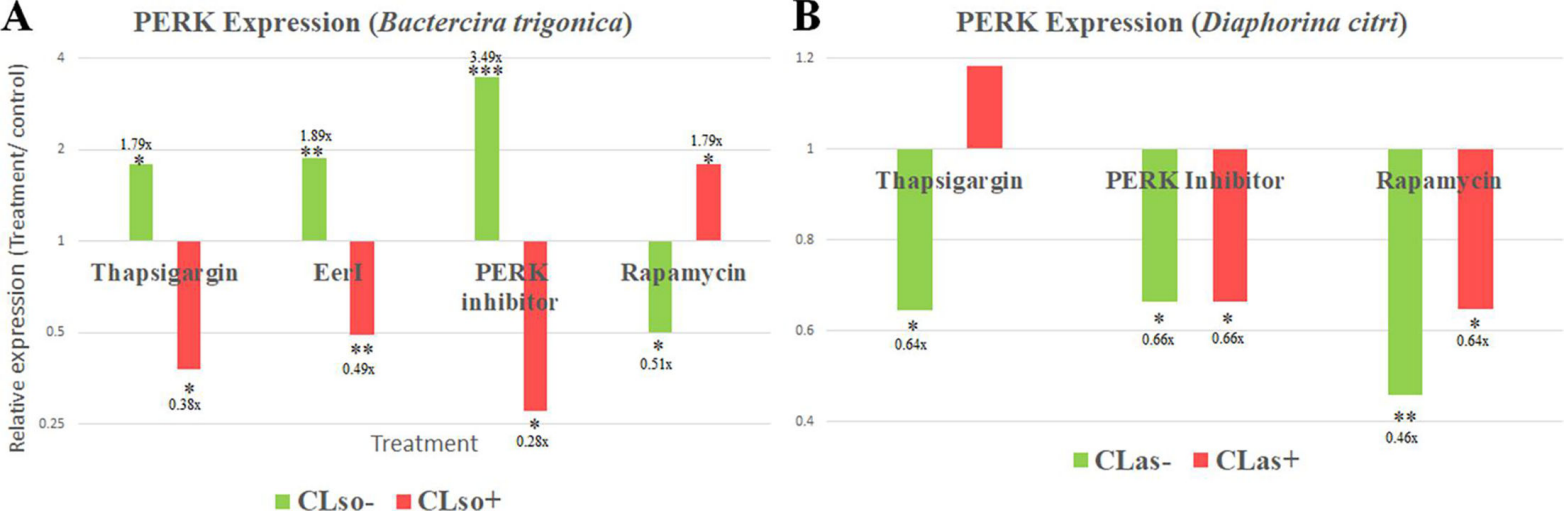
PERK expression in CLso/CLas-infected and uninfected psyllids after different chemical treatments. The relative expression of PERK in the carrot (**A**) and citrus (**B**) CLso/CLas-infected and uninfected psyllids after treating them with three chemical stressors and rapamycin (an autophagy inducer). 10 µM of each treatment for CLso/CLas-infected psyllids or 40 µM for uninfected samples were used. (*P < 0.05, **P < 0.01, ***P < 0.0001).

Inhibiting PERK using specific inhibitors in CLso-uninfected psyllids increased PERK expression, while the expression decreased after inhibition in CLso-infected psyllids (Fig. 5A). Inducing ER stress using other drugs (thapsigargin, Eeyaristatin I, and rapamycin) resulted in different expression profiles in CLso-infected and uninfected psyllids (Fig. 5A). The expression of PERK was also measured in CLas-infected and uninfected *D. citri* after PERK inhibitor I, thapsigargin, and rapamycin treatments. Interestingly, the results with CLas were different compared with CLso. The effect of rapamycin and PERK inhibitor I treatments on the expression of PERK was not influenced by CLas infection, while thapsigargin resulted in opposite results compared to CLso-*B. trigonica* system. (Fig. 5B).

### ER stress drugs differentially influence CLso and CLas titers

**Fig 6:**
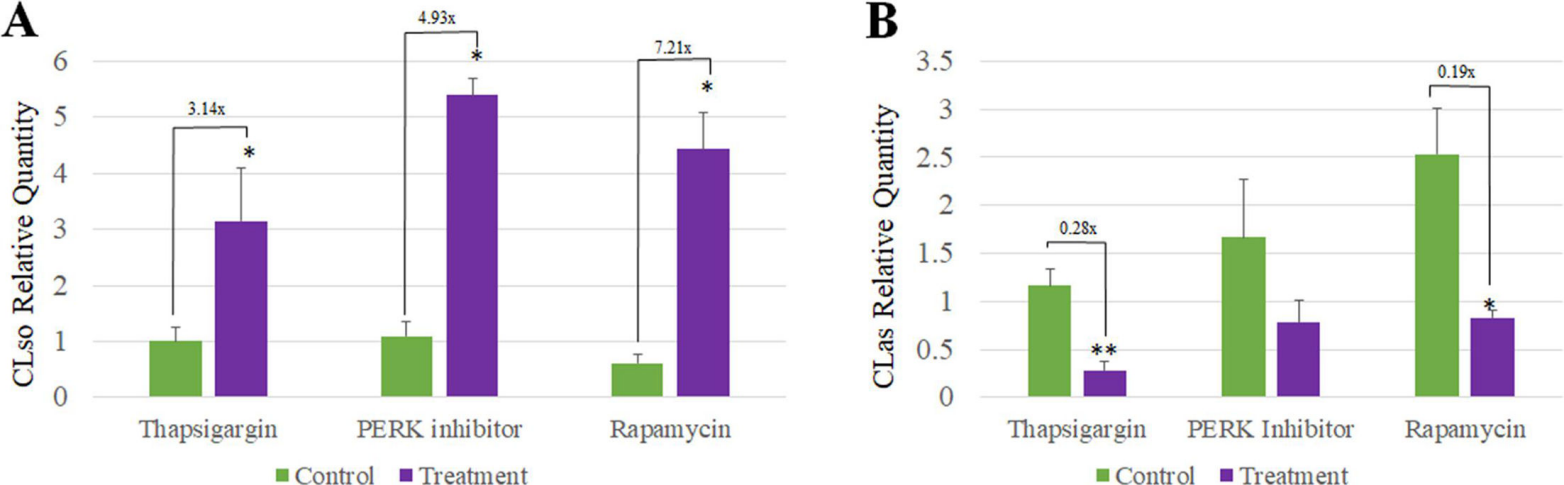
CLso and CLas titers in the midguts of the psyllids after different chemical treatments. The relative quantities of CLso in *B. trigonica* (**A**) or CLas in *D. citri* (**B**) were quantified after three different chemical treatments. Showing that the same treatments had differentially influenced the two systems. (*P < 0.05, **P < 0.01).

To test whether CLso levels in psyllid midguts are influenced by drug treatments, DNA was extracted from midguts of CLso-infected psyllids of both control and treatment from the same experiments described in Fig. 5. qPCR results showed that, similar to the increased CLso titers observed after Eeyaristatin I treatment (19), CLso titers in midguts were significantly induced following PERK inhibitor I, thapsigargin, and rapamycin treatments (Fig. 6A). Surprisingly, conducting a similar experiment in the *D. citri* and CLas system showed that, unlike CLso, the levels of CLas were decreased (Fig. 6B).

**Fig 7:**
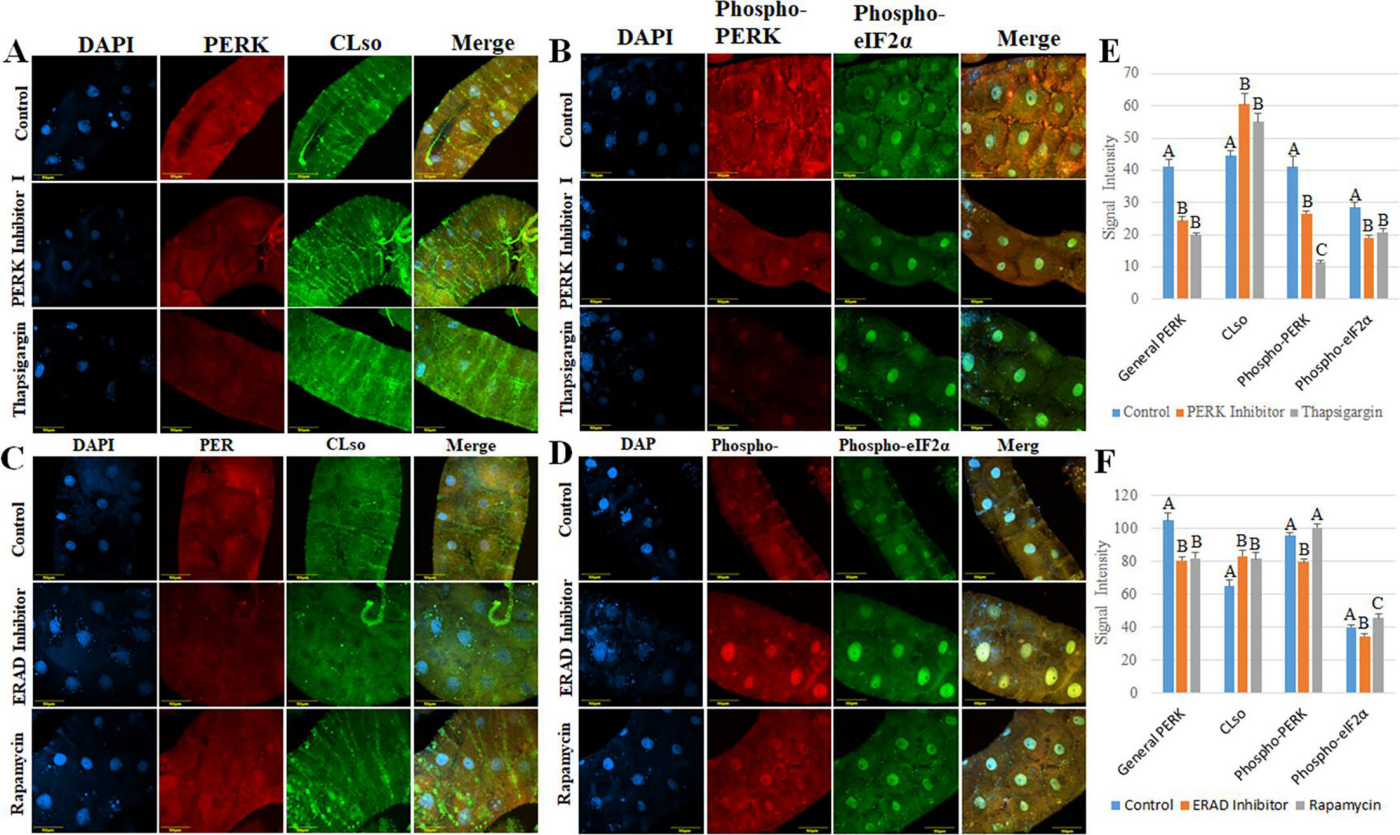
Immunostaining of CLso, PERK, phospho-PERK, and Phospho-eIF2α after the chemical treatments. Co-immunostaining of CLso (green) and PERK (red) in midguts dissected from control, PERK inhibitor I, and thapsigargin (**A**), or Eeyaristatin I and rapamycin (**C**). Co-immunostaining of phosphorylated PERK (phospho-PERK) (red) and phosphorylated eIF2α (phospho-eIF2α) (green) in midguts dissected from control, PERK inhibitor, and thapsigargin (**B**), or Eeyaristatin I and rapamycin treated psyllids (**D**). All samples were counterstained with DAPI (blue). (**E, F**) Significant differences in the intensity of CLso, PERK, phospho-PERK, and phospho-eIF2α after the different treatments were measured using FIJI software. Different letters indicate statistical differences at *P* < 0.05 using Tukey–Kramer Honest Significant Difference (HSD) test.

To confirm the qRT-PCR results, immunostaining using Anti-CLso and Anti-PERK antibodies was performed after treating with the same drugs (Fig. 7A and C). The signal intensity levels were quantified using Fiji software, which further confirmed the qRT-PCR results (Fig. 7E and F). Similar to qPCR results, CLso amounts were increased after all three treatments, while PERK levels decreased.

### Expression of the active PERK after drug treatments

To test whether the drug treatments influence the expression of the active form of PERK, immunostaining was performed. The amount of phosphorylated PERK and eIF2α was measured in the midguts after drug treatments as described above (Fig. 7B and D). Results show that the active form of PERK correlates with its expression (Fig. 7A). The signal intensity levels were quantified using Fiji software, which further confirmed the qRT-PCR results (Fig. 7E and F). The amount of Phospo-PERK and Phospho- eIF2α decreased after thapsigargin and Eeyaristatin I treatments, while it increased after rapamycin treatment.

### PERK inhibition influences the expression of UPR, ERAD, and apoptosis-related genes

**Fig 8:**
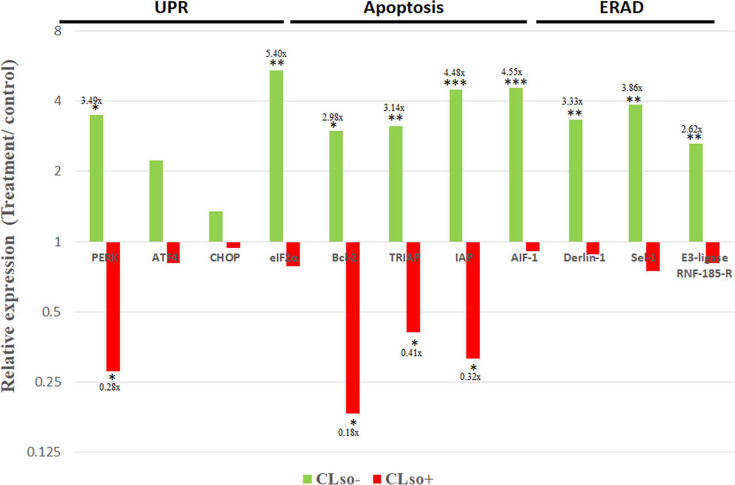
Expression profile of genes related to UPR, apoptosis, and ERAD in CLso-infected and uninfected psyllids after PERK inhibitor treatment. 10 µM of the treatment for CLso-infected psyllids or 40 µM for uninfected samples were used. (*P < 0.05, **P < 0.01, ***P < 0.0001).

To further investigate the involvement of PERK and CLso in the observed induced apoptosis, the expressions of genes related to UPR (PERK pathway), ERAD, and apoptosis were quantified after PERK inhibition. As shown in Fig. 8, the expression of PERK was significantly affected by inhibitor treatment (as shown in the previous experiments) in both CLso-infected and uninfected psyllids, but no significant changes were observed in the expression of eIF2α, ATF4, and CHOP in CLso-infected samples. In CLso-uninfected psyllids, the inhibitor increased the expression of the PERK pathway, though it was significant only for eIf2α. The treatment resulted in inducing apoptosis in CLso-infected psyllids, while it was reduced in CLso-uninfected ones. No significant effect on ERAD gene expression was obtained following the same treatment in CLso-infected psyllids, while their expression was upregulated in CLso uninfected ones.

### UPR, ERAD, and apoptosis-related genes expression after ER stress induction

To test if the observed differential effect on CLso-infected and uninfected psyllids was due to ER stress in general or if it was specific for inhibiting PERK, the expression of the same genes was quantified following thapsigargin treatment, which specifically induces ER stress (Fig. S2). Thapsigargin treatment did not significantly alter the expression of UPR genes, except for PERK, as previously shown (Fig. 5A). A modest, non-significant decrease in apoptosis inhibitor expression was observed in both CLso-infected and uninfected psyllids, with a weaker effect compared to PERK inhibitor I (Fig. 8). In the case of the ERAD pathway, opposite results were observed. ERAD was induced after thapsigargin treatment in CLso-infected psyllids, whereas it was not significantly affected in CLso-uninfected ones.

## DISCUSSION

Previous reports have shown that *Candidatus Liberibacter asiaticus* (CLas) induces apoptosis in the midgut of ACP (34). In contrast, no such evidence was observed in the potato psyllid *Bactericera cockerelli* to CLso infection (41, 42). In this manuscript, we used FISH and quantified nuclear disruption, an indication of apoptosis, in CLso-infected and uninfected midguts of the carrot psyllid using a CLso-specific probe and DAPI staining (Fig. 2B). The results showed higher levels of disruption in CLso-infected midguts (73.3%) compared to the significantly lower disruption levels in psyllids reared on healthy plants (20.8%). Quantifying the expression of apoptosis-related genes in CLso-infected and uninfected adult psyllids and midguts confirmed that apoptosis is likely to be induced in CLso-infected samples (Fig. S1).

Previous studies have used electron and fluorescent microscopy and showed that *Liberibacter* is associated with the ER in midgut cells (37). In these studies, it was confirmed that CLas recruits ER-associated structures and forms *Liberibacter*-containing vacuoles (LCVs), which the bacterium uses for replication, thus avoiding the cellular immune responses in the insect midgut. We have also shown that CLso induces the ER-associated degradation (ERAD) machinery in the psyllid’s midgut (38). Additionally, Derlin-1, a key ERAD gene, was associated with CLso retention and stability in psyllid midgut cells and significantly influenced its transmission (19).

ER homeostasis is constantly challenged by physiological demands and external stress factors, such as infection with pathogens. Three pathways of quality control are initiated in the ER and are responsible for maintaining ER homeostasis: the UPR, ERAD, and autophagy (43, 44). ERAD is responsible for the recognition and disposal of misfolded proteins in the ER lumen, while autophagy is responsible for degrading protein aggregates. Failure to degrade misfolded proteins in the ER triggers UPR, a coordinated response whose combined effect is the upregulation of genes encoding proteins involved in the secretory pathway, aiming to help the cell cope with ER stress (See Fig. 1). UPR increases the ER protein-folding capacity, reduces global protein synthesis, and enhances both ERAD of misfolded proteins and autophagy (20, 22). If ER stress is prolonged and unresolved, UPR downstream pathways lead to apoptosis. The combined response of the three ER stress sensors contributes to either adaptation or apoptosis. The observed induction of apoptosis in CLso-infected psyllids can be associated with ER stress triggered by CLso and mediated through UPR. Among the three sensors, we were able to recover and identify the sequence of only PERK and IRE1 using deep sequencing (38). Since PERK is a central component of the UPR and is typically activated under ER stress to mediate downstream pro-apoptotic responses, we expected its expression to increase in CLso-infected psyllids. Surprisingly, gene expression analysis in CLso-infected and uninfected adult psyllids and midguts showed that PERK is downregulated in CLso-infected samples (Fig. 3B), while no significant difference in the expression of key PERK pathway genes was obtained (not shown). PERK downregulation was confirmed using immunostaining (Fig. 3A and C), suggesting that PERK transcript levels do not reflect its activation status. PERK upregulation was demonstrated after infection with *Francisella tularensis*, the causative agent of Tularemia, while its activity was found to be attenuated (45). To test the expression of the activated form of PERK in CLso-infected psyllids, the levels of phosphorylated PERK and eIF2α, which is directly phosphorylated by PERK (see Fig. 1), were visualized and quantified using specific antibodies. Despite the downregulation of PERK following gene expression analysis, both phosphorylated PERK and eIF2α accumulated in CLso-infected midguts compared to the uninfected ones (Fig. 4A and C). The immunostaining results indicate that the PERK pathway is activated in midgut cells following CLso infection. Previous reports have shown that IRE1 and ATF6 can be attenuated by persistent ER stress, while PERK signaling is sustained (30, 46). Continued PERK signaling impairs cell proliferation and promotes apoptosis by the PERK-ATF4-CHOP pathway. Some pathogens manipulate or selectively induce UPR pathways to promote their growth and survival. For example*, Francisella tularensis* modulates the expression of BiP, increasing the activation of IRE1α and ATF6 while decreasing PERK phosphorylation and CHOP expression (45). *Chikungunya*, a mosquito-transmitted virus, activates ATF6 and IRE1α branches of the UPR but blocks PERK signaling by suppressing eIF2α phosphorylation (47, 48). The PERK pathway can also regulate virus replication by attenuating the global protein synthesis in the host cells in case of ER stress. As mentioned earlier, the expression of ATF4 and CHOP was not significantly affected by CLso infection (not shown), even though PERK activity is induced. Similarly, infection with *Legionella* and other microbial pathogens disrupts the apoptotic response of UPR by specifically attenuating the production of ATF4 and CHOP in the PERK pathway (49, 50).

To further investigate the association of PERK with CLso and its involvement in the induced apoptosis, different drugs were used. The expression of PERK was quantified after treating the insects with three different ER stressors (thapsigargin, Eeyaristatin I, and PERK inhibitor I) and rapamycin, a known autophagy inducer, in both CLso-infected and uninfected psyllids. Initially, a concentration of 10 µM was used for both CLso-infected and uninfected psyllids. While this concentration was effective in CLso-infected psyllids, it did not induce significant changes in the uninfected samples. This lack of response was likely due to the absence of pre-existing ER stress/induced autophagy in the uninfected psyllids, making the lower treatment insufficient to induce a measurable effect. To address this, we increased the concentration to 40 µM for the uninfected psyllids, resulting in measurable changes in gene expression. The effect on the expression of PERK after all four drug treatments was noticeably different in CLso-infected psyllids compared to the healthy ones (Fig. 5A). As mentioned earlier, three of the drug treatments are ER stressors, and since inducing ER stress activates UPR, in CLso-uninfected psyllids, as predicted, PERK was upregulated after all three treatments, whereas in CLso-infected psyllids, significantly different results were obtained. Inducing ER stress by adding additional stress with CLso caused a further decrease in PERK expression. Rapamycin treatment also showed contrasting effects on PERK expression in the two populations. In CLso-uninfected psyllids, PERK expression was downregulated, while in the infected psyllids, it was upregulated. Rapamycin induces autophagy by inhibiting the mechanistic target of rapamycin complex 1 (mTORC1), which can alleviate ER stress and subsequently downregulate PERK expression and activity (51–53). This effect was evident in the CLso-uninfected psyllids, where no pre-existing ER stress was present (Fig. 5A). However, the effects of rapamycin on ER stress and PERK pathway are complex and context-dependent. While rapamycin-induced autophagy can mitigate ER stress under certain conditions, in other scenarios, particularly in the presence of pre-existing ER stress, it may be insufficient to fully resolve the stress (54). This can lead to sustained or even increased PERK activation, which explains the observed upregulation of PERK in the infected psyllids. Interestingly, quantifying the expression of PERK after PERK inhibitor I and thapsigargin and rapamycin treatments in CLas-infected and uninfected *D. citri* showed different results (Fig. 5B). In CLas-*D. citri* system, inhibiting PERK resulted in a similar effect across both infected and uninfected psyllids, which was unrelated to the bacterium infection. Applying thapsigargin resulted in an opposite effect compared with CLso-*B. trigonica* system. CLso titers in the midguts were also quantified after drug treatments, and the results were similar to our previously published results with CLso after Eeyaristatin I treatment (19), in which treating psyllids with PERK inhibitor I, thapsigargin, and rapamycin increased CLso titers in the midguts (Fig. 6A). The same effect was not observed in the CLas-*D. citri* system (Fig. 6B), in which such drug treatments decreased the titers, suggesting that PERK-*Liberibacter* interactions are likely to be different in the CLas system. Regarding rapamycin treatment, the results obtained from this study are not comparable to a previous study in which rapamycin reduced CLso titers in the psyllids midguts (55). Two hypotheses might explain these results. First, inducing autophagy can enhance *Liberibacter* survival at certain stages of infection, as suggested for CLas and other pathogens, in which hijack autophagic components from hosts to form autophagosome-like compartments as replication niches for their growth and intercellular spread, thereby escaping host defenses, enabling transmission and release, and a process that has been associated with the ER (56–59). Another possible explanation is that the rapamycin effect is likely to be stage-dependent or titer-dependent. Higher pathogen loads and the associated ER stress might modulate the outcome of rapamycin treatment, leading to sustained PERK activation and enhanced bacterial proliferation.

Increase in CLso levels after inhibiting PERK suggests that PERK has a significant effect on the bacterium. Using specific antibodies against PERK, CLso, and the phosphorylated forms of PERK and eIF2α after drug treatments allowed detection of these proteins, and the levels of CLso and PERK (Fig. 7A and C) confirmed the qRT-PCR results. Detecting phosphorylated PERK and eIF2α was in correlation with the results of the expression of PERK gene and protein expression (Fig. 7B, D–F). Our results also support a strong association between PERK activity and CLso-induced apoptosis, as shown by the differential expression of key genes involved in UPR, apoptosis, and ERAD following PERK inhibition in CLso-infected and uninfected psyllids (Fig. 8). The expression of all the tested genes was different in CLso-infected psyllids compared to the controls, a result that further confirmed the interaction between PERK and CLso, as well as the ability of the bacterium to influence the expression of PERK. Treatment with thapsigargin, which is widely used as a positive control for UPR (60), and measuring the expressions of the same genes was conducted to test whether the response observed was a specific response after inhibiting PERK or a response following general ER stress (Fig. S2). The results indicated that thapsigargin had varying effects on the tested genes of the three pathways, suggesting that the expression profiles observed after PERK inhibition were a specific response to inhibiting PERK and not a result of general ER stress.

In conclusion, similar to previously published reports about the psyllid’s immune response to *Liberibacter* infection (34, 40), this study experimentally confirms that apoptosis is induced in *B. trigonica* as a result of CLso infection. The activity of PERK, one of the three UPR sensors, is induced in the midguts of CLso-infected psyllids, although the expression of the gene was significantly downregulated. Infection with CLso influences the expression profile of PERK after treating with ER stressors. Similar results were not observed in the case of the CLas-D. *citri* system, suggesting that the interactions between ER, UPR, and *Liberibacter* are different between the two systems. In light of these results, the PERK pathway and UPR appear to be involved in the apoptotic response associated with CLso infection. Their exact role is yet to be determined; however, the current findings are a significant step in understanding the interaction between the ER, associated stress pathways, and CLso in the psyllids midgut. These results are important for studying and understanding other pathogen-vector systems in plant and animal pathogens that are vector-borne, as well as the involvement of the immune system in their retention, replication, and transmission.

## MATERIALS AND METHODS

### Insect colonies

CLso-infected and uninfected psyllid populations were reared on parsley plants and maintained inside insect-proof cages in separate climate-controlled growth rooms at 25 ± 2°C with a photoperiod of 16:8 h. Parsley plants were grown in a potting mix in 1.5 L pots under artificial light and maintained inside a clean room in a glasshouse under the same controlled conditions. Experiments typically involved adult psyllids aged 5–10 days, unless noted. Late-stage (4th–5th instar) nymphs were used for all nymphal experiments.

### RNA extraction from whole adults/midguts and qRT-PCR

RNA extraction from midguts or whole adults using Tri reagent (Sigma Aldrich) was performed using a recently published protocol (19). Briefly, psyllids or midguts were homogenized in Tri reagent using a micro-pestle. 0.2 volumes of chloroform were added and centrifuged at 12,000 rpm for 15 min at 4°C for phase separation. The upper aqueous phase was transferred to a fresh tube, gently mixed with 0.7 volumes of isopropanol, and incubated overnight at −20°C for precipitation. The samples were then centrifuged at 12,000 rpm for 30 min at 4°C. The supernatant was removed, and the pellet was washed with 75% ice-cold ethanol. The pellet was dissolved in double-distilled water. DNA contaminations were removed using DNase I (Thermo Scientific). RNA purity and yield were analyzed using a NanoDrop1000 spectrophotometer (Thermo Fisher Scientific) and were kept in −80°C until further use. cDNA was synthesized using M-MLV reverse transcriptase (Promega Corporations) following the manufacturer’s instructions. To quantify expression levels of the psyllid genes in whole adults and midguts, quantitative RT-PCR (qRT-PCR) was carried out using ABsolute Blue SYBR Green mix on a StepOne real-time PCR system (Applied Biosystems). Primers were designed from the sequences derived from the psyllid transcriptomic data (38). Ct values were normalized using the Actin gene (Table S1). The expression of each gene was calculated by the delta-delta Ct method (−2^ΔΔCT^) (61). Each experiment included three biological replicates, with three technical replicates per sample, and was repeated three times.

### DNA extraction from psyllids midguts and quantification of CLso (qPCR)

For DNA extraction from single midguts, Chelex 100 was used (62) with slight modifications. Each gut was homogenized in 100 μL TE solution (10 mM Tris–HCl and 1 mM EDTA, pH 8.0) containing 10% Chelex (Bio Rad, USA) and 300 μg proteinase K. The samples were incubated at 37°C for 1 h, followed by protein denaturation at 95°C for 10 min and centrifugation at 10,000 rpm for 5 min. The aqueous phase was transferred to a clean tube. DNA purity and yield were analyzed using a NanoDrop 1000 spectrophotometer and were kept in −20°C until further use. Real-time analysis (q-PCR) was carried out as described previously, using the elongation factor gene sequence as a housekeeping gene (Table S1). Each experiment included a minimum of three biological replicates, with three technical replicates per sample, and was repeated three times.

### Fluorescent *in situ* hybridization (FISH)

Midguts from adults and nymphs of healthy and CLso-infected psyllids were dissected in 1× PBS (phosphate-buffered saline, pH 7.2) inside a depressed glass well, followed by fixation with Carnoy’s fixative (chloroform: ethanol: glacial acetic acid [6:3:1]) for 5 min. Then, they were washed with hybridization buffer (20 mM Tris HCl [pH 8], 0.9 M NaCl, 0.01% SDS, 30% formamide) and incubated with 10 pmol Cy3-labeled fluorescent probe/mL for the outer membrane protein B of CLso (Table S1). The midguts were then washed again with hybridization buffer, mounted with 4′, 6-diamidino-2-phenylindole (DAPI) in hybridization buffer (0.1 mg/mL), and viewed using a confocal microscope (Olympus IX81). Sixteen images from each population were analyzed.

### Immunostaining

Immunostaining for PERK, CLso, phosphorylated PERK, and eIF2α was performed according to the protocol previously described (19). Dissected psyllid midguts in PBS were fixed for 1 h with 4% paraformaldehyde, followed by treatment with 0.01% Triton X-100 for 30 min. Samples were then washed three times with PBST and blocked using 1% blocking buffer (bovine serum albumin) for 2 h at room temperature. The midguts were then incubated with anti-outer membrane protein B antibody for CLso (GenScript Corp., USA; dilution 1:2,000) and anti-PERK antibody (Santa Cruz Biotechnology; 1:50), or, to check the activated proteins, anti-phospho-PERK (Abcam; 1:200) and anti-phospho-eIF2α (Sigma Aldrich; 1:200), followed by secondary antibody conjugated with Cy3/Cy5 (Jackson Immuno-Research Laboratories; 1:1,000). Samples were then washed three times with PBST, mounted with DAPI (0.1 mg/mL), and viewed using a confocal microscope (Olympus IX81). The intensity of the fluorescence signals was quantified and validated using FIJI software (63). A minimum of five images were analyzed for each population/treatment.

### Investigating the role of PERK in CLso-ER interactions using drug treatments

Different drug treatments were used to investigate the role of PERK in CLso interactions with the ER of psyllid cells. In these experiments, CLso titers and PERK expression and activity were measured. The experiments were performed by soaking parsley stems in 10 µM for CLso-infected psyllids or 40 µM for uninfected samples of thapsigargin (ER stressor and apoptosis inducer), Eeyaristatin I (ER-associated degradation (ERAD) inhibitor), PERK inhibitor I, or rapamycin (autophagy inducer) for 24 h. This was followed by releasing CLso-infected and uninfected psyllids inside a glass jar sealed with a breathing cloth for 48 h. Live insects were collected and used for RNA (three whole adults per sample)/DNA (one midgut per sample) extraction for quantifying the expression of UPR, apoptosis, and ERAD-related genes and the titers of CLso, respectively, or for immunostaining. The drug treatments used are known to induce ER stress in different manners: PERK inhibitor I (GSK2606414, Sigma, dissolved in DMSO) binds to PERK at the ATP-binding region and prevents its activation (64). Thapsigargin (Santa Cruz, dissolved in ethanol) blocks the sarcoplasmic/endoplasmic reticulum Ca^2+^-ATPase (SERCA) pump and rapidly depletes ER Ca^2+^, inducing ER stress and eventually leading to induced apoptosis via UPR (65, 66). Eeyaristatin I inhibits ERAD by targeting the ER-associated p97 ATPase, causing downstream block of the retro-translocation and de-ubiquitination process (67). Rapamycin (Sigma, dissolved in ethanol), a specific inhibitor of mTORC1, a protein complex involved in the ER stress response and the regulation of cell fate decisions (52, 53, 68). For each treatment, control plants were soaked in water containing the same concentration of solvent as the chemical treatments. The expression of PERK and the titers of CLas were measured after conducting similar experiments using three of the treatments with ACP. Each experiment included a minimum of three biological replicates, with three technical replicates per sample, and was repeated three times.

### Statistical analysis

The significance of the differences between means in all comparisons, performed on data from the qRT-PCR/qPCR assays and FIJI software measurements, was analyzed using the one-way analysis of variance (ANOVA) or Tukey–Kramer Honest Significance Difference (HSD) test (α = 0.05). JMP Pro software (SAS Institute) was used for the analyses.

## SUPPLEMENTAL MATERIAL

Supplemental material spectrum.03025-25-s0001.pdf Fig. S1 and S2; Table S1.

ASM does not own the copyrights to Supplemental Material that may be linked to, or accessed through, an article. The authors have granted ASM a non-exclusive, world-wide license to publish the Supplemental Material files. Please contact the corresponding author directly for reuse.
