# Supplementary material for: Protein kinase R-like ER kinase (PERK) is involved in the endoplasmic reticulum stress response of its psyllid vector to Candidatus Liberibacter solanacearum infection
Source: Microbiol Spectr. 2025 Dec 26;14(2):e03025-25. doi: 10.1128/spectrum.03025-25 (PMC12889044; doi:10.1128/spectrum.03025-25)
Supplement: Supplemental material — Fig. S1 and S2; Table S1. [file spectrum.03025-25-s0001.pdf]

Supplemental material

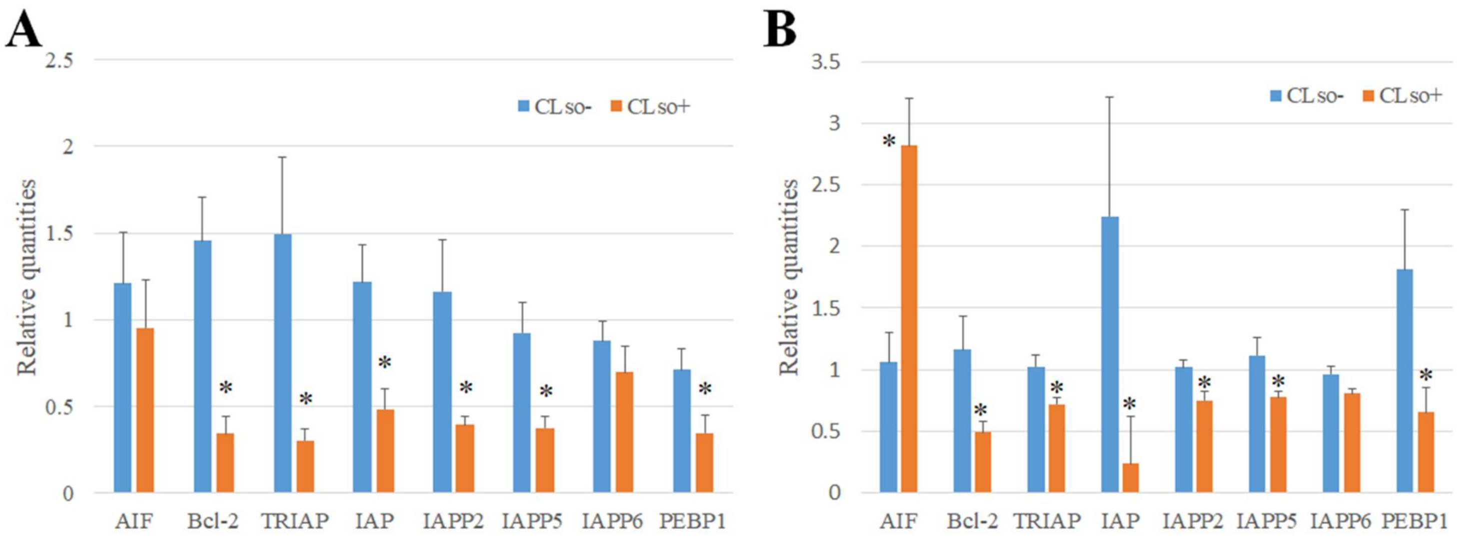

Fig. S1 Relative quantification of apoptosis-related genes. Apoptosis-related genes expression profile in midguts (A) and whole adults (B) of CLso-infected and uninfected psyllids shows induced apoptosis in CLso-infected samples. (*$P<0.05$)

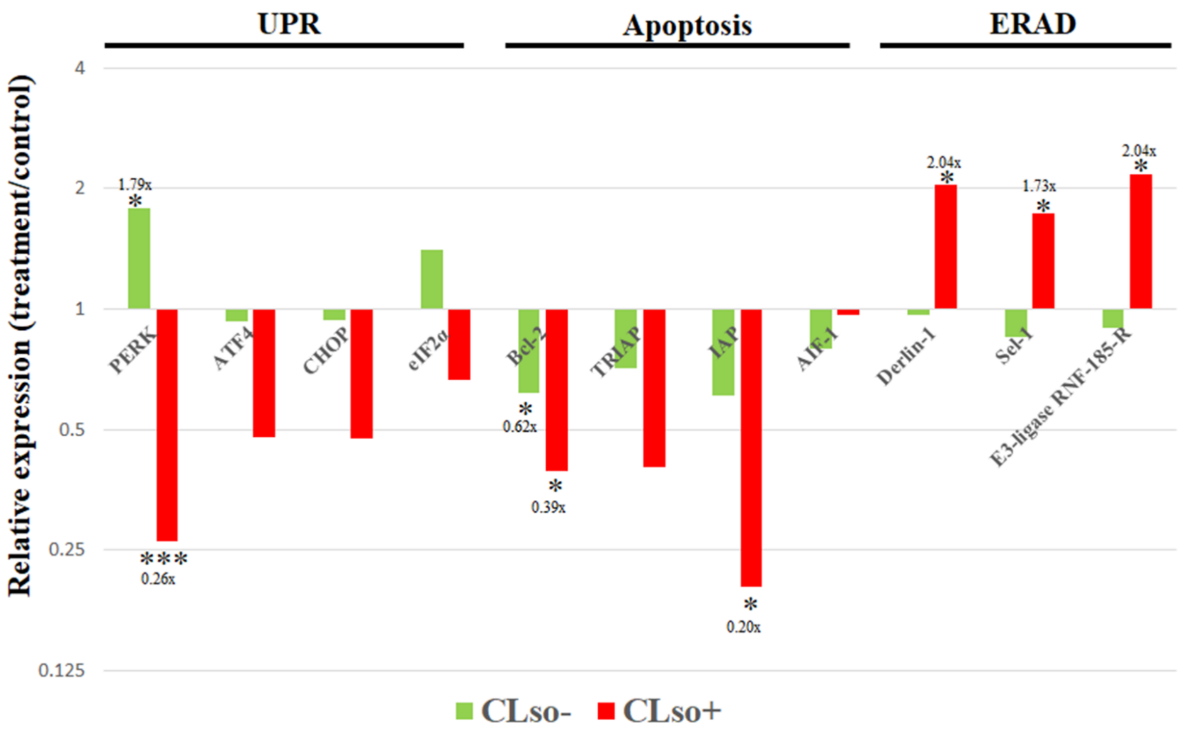

Fig. S2 Expression profile of genes related to UPR, Apoptosis, and ERAD in CLso-infected and uninfected psyllids after using a different ER stressor (thapsigargin). 10 µM of the treatment for CLso-infected psyllids or 40 µM for uninfected samples were used. (*P<0.05, **P<0.01, ***P<0.0001)

Table S1. Primer sequences and probes used in this study

Name	Sequence (5′ to 3′)	Product size (bp)	Reference
q-Liberibacter-F (CLso)	CCATATCCAAATTTCAAAGAACC	152	(69)
q-Liberibacter-F (CLso)	ATGCCACGTGAAGGTTTGAT		
Actin-F	AGATGACCCAGATCATGTTTGA	140	(69)
Actin-R	AGGGCGTAACCTTCATAGATG		
PERK-F	TCCAGGAGGTAAAAGCTCGC	148	This study
PERK-R	TCAGAATCGGAAGCGGAGGA		
eIF2a-F	AGTATCTCAGCCACATGCCG	207	This study
eIF2a-R	GCTGTCCAGGAGGCGAATTA		
ATF4-F	GAACAGAACAAGAATGCCGC	206	This study
ATF4-R	CGGGCCGGATATTACTTGAG		
CHOP-F	CGCGAGCGAAATAACATAGC	195	This study
CHOP-R	ATGTAAGTGCTCGGGAAGGA		
Derlin-1-F	GGATGGTGTGGCCAGTAA TG	174	(38)
Derlin-1-R	CGCACACAGTTCAAAGCA ATG		
Sel-1-F	ATTGCTCATCTCTTGGCTTGA	167	(38)
Sel-1-R	GCAAACTTCTAACCGAGCCT		
E3-ligase RNF-185-F	GCAAATACCACCGAACAGAGA	147	(38)
E3-ligase RNF-185-R	GACAGGGCCAACAGAATAAGT		
Elongation Factor, Exon (EF-EX)-F	TTGATGACTCCCACAGCCAC	90	(19)
Elongation Factor, Exon (EF-EX)-F	CATGTGCGTCGAGTCCTTCT		
Bcl2-F	CAGAACTTGAAAGGACCGCC	110	This study
Bcl2-R	GATGTTCCCTTCGGACATGC		
TRIAP1-F	GGAATGCACACAGCTCAAACA	112	This study
TRIAP1-R	AACCCTCAGCAACGGAGAAC		

AIF1-F<br>AIF1-R	AGCCCGAAGACTTCTACACC<br>CCAGCCTCGTACAACTGAGA	188	This study
IAP-F<br>IAP-R	GAGTCAAATGATCGCACTCG<br>TCTACTGATACAACCCCCGA	165	This study
IAPP2-F<br>IAPP2-R	TTGCAAGCCTACCAGACTCG<br>AGAATTGCCAAGGCAGGGTT	220	This study
IAPP5-F<br>IAPP5-R	TTCTTTCCACGGGTCATCGG<br>TTCAAGGATTGGCCGTTCAGA	150	This study
IAPP6-F<br>IAPP6-R	AGCAAGCTCTGTTGGGTTCT<br>AGGTGCACTACTAAGGGGCT	215	This study
PEBP-F<br>PEBP-R	TAGTGCGTCTTTTAAGCGGG<br>TCGTTCGTTAGAGCTGTTGG	199	This study
ACP-GAPDH-F<br>ACP-GAPDH-R	GACACTCACTCCTCCATCTTT<br>GTATCCGTACTCGTTGTCATACC	88	(70)
PERK-ACP-F<br>PERK-ACP-R	CCTGGCTAGAGTGTCCTCCA<br>GTGAGGTCGGCAGAGGTTTG	115	This study
CLas-CQ-F<br>CLas-CQ-R	TGGAGGTGTAAAAGTTGCCAAA<br>CCAACGAAAAGATCAGATATTCCTCTA	87	(56)
CLso probe	5'- CY3-GCCTCGCGACTTCGCAACCAAT -3'		(71)

References cited only in the supplemental materials:

69. Ghosh S, Sela N, Kontsedalov S, Lebedev G, Haines LR, Ghanim M. An intranuclear Sodalis-like symbiont and Spiroplasma coinfect the carrot psyllid, Bactericera trigonica (Hemiptera, Psylloidea). Microorganisms. 2020;8(5):692.

Formatted: Justified

70. Wang Z, Yin Y, Hu H, Yuan Q, Peng G, Xia Y. Development and application of molecular-based diagnosis for ‘Candidatus Liberibacter asiaticus’, the causal pathogen of citrus huanglongbing. Plant Pathol. 2006;55(5):630–8.

71. Munyaneza JE, Sengoda VG, Crosslin JM, De la Rosa-Lozano G, Sanchez A. First report of ‘Candidatus Liberibacter psyllaurous’ in potato tubers with zebra chip disease in Mexico. Plant Dis. 2009;93(5):552.
